# The evolving CRISPR technology

**DOI:** 10.1007/s13238-019-0645-9

**Published:** 2019-06-26

**Authors:** Meng Yan, Jinsong Li

**Affiliations:** State Key Laboratory of Cell Biology, Shanghai Key Laboratory of Molecular Andrology, CAS Center for Excellence in Molecular Cell Science, Shanghai Institute of Biochemistry and Cell Biology, Chinese Academy of Sciences, University of Chinese Academy of Sciences, Shanghai, 200031 China

**Figure 1:**
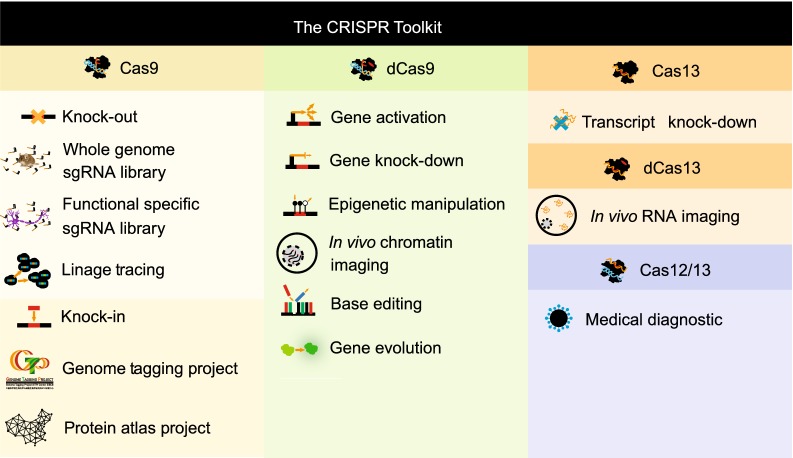
The CRISPR toolkit

Advances in genome editing technology have revolutionized basic and applied biology research in recent years, particularly due to the newly emerged CRISPR/Cas technique (Ren et al., 2017b). The classical CRISPR/Cas system was derived from a bacterial defense system, which consists of a single guide RNA (sgRNA) for precise targeting and one Cas protein for DNA binding and nuclease activity. Relying on high precision of CRISPR/Cas system, multiple functions have been exploited in addition to original nuclease activity, such as genome base editing, gene knock-down and activation, chromatin imaging systems etc. (Barrangou and Doudna, 2016; Wang and Qi, 2016). These extensions have demonstrated the flexibility of the CRISPR/Cas system as it has boosted basic biological, biomedical and agricultural research (Shen et al., 2017; Zhang et al., 2017). Here we summarize some key events in CRISPR/Cas system development, provide a glimpse of how this technology has transformed biological research, and help you to choose the right tool for your own research (Fig. 1).

CRISPR/Cas system was first introduced into human 293FT cells where it was shown to cleave target sites with high efficiency. The resulting double-stranded breaks (DSBs) in genomic DNA were partially repaired by the indel-forming nonhomologous end joining (NHEJ) pathway (Cong et al., 2013); therefore indels (insertions/deletions) and base mutations may present within the target site, which could deactivate the target gene and achieve *in vivo* gene knock-out. The convenience and precision of this technique has facilitated its rapid adoption to the genome of a wide range of species, including human, monkey, mouse, rat, fly, zebrafish, *Arabidopsis Thaliana* and rice and so on. Because its targeting precision and efficiency have been well studied, targeting sites can be designed to minimize off-target binding and maximize on-target efficiency (Hsu et al., 2013). A recent research identified an advanced xCas9 variant with broad protospacer adjacent motif (PAM) compatibility and high DNA specificity, suggesting that higher editing efficiency, better PAM compatibility and DNA specificity can be achieved at the same time (Hu et al., 2018). In addition, based on the structure of Cas9, rationally engineered SpCas9-NG variant, also with an increased targeting range (higher activity towards NGD sites than NGC sites), promises a better efficiency over xCas9 at NGD targeting sites and suggests a way to design Cas9 for further optimization (Nishimasu et al., 2018).

Instead of a single sgRNA, a well-purposed sgRNA library could facilitate large-scale functional genomic studies (Shalem et al., 2014; Zhong et al., 2015), providing a competitive approach to screen key components in biological processes of interest. Tissue-specific, cancer-related, and whole genome-scale libraries have been built to study underlying biological pathways and drug resistance. The application of the low-cost, highly convenient and flexible CRISPR/Cas systems to medical research enables large-scale screenings that reduces total investment in research and development without compromising outcomes.

Homology-directed repair (HDR) pathway offers another option for cells to repair DSBs (instead of NHEJ). When a DNA template with a flanking homologous sequence is introduced, cells can repair DSBs with precise recombination at the DSBs site (CRISPR/Cas knock-in system). Utilizing the concept, we could repair the mutations that induce diesease, which propose a bright future in gene therapy (Wu et al., 2013; Wu et al., 2015). In the meantime, to study pathology of human genetic diseases, especially polygenic diseases, we could mimic the disease mutations in model organisms easily by using this method, which, otherwise, might be extremely difficult to achieve by other methods. Take one step further, the concept could also be used to add a tag to a target, protein or RNA molecule. It has been shown that mouse models can be generated using “artificial spermatids” (Yang et al., 2012), by integrating with CRISPR/Cas knock-in system, the recently launched genome tagging project (GTP) will provide *in vivo* tagged mouse models at a researcher’s request (Jiang et al., 2018). This project shows versatility and capability of CRISPR systems and brings *in vivo* research from the cellular level to the animal level. Based on GTP, a new project, termed protein atlas project (PAP), may be proposed to extend the horizon of GTP by focusing on the *in vivo* protein–protein interaction networks and spatiotemporal expression patterns on a large scale.

Over the past several years, great efforts have been made to innovate in the CRISPR/Cas system, including gene knock-down, activation, evolution, *in vivo* chromatin imaging, epigenetic modification, base editing and lineage tracing systems. These CRISPR/Cas subsidiaries have promoted multi-dimensional applications of original CRISPR/Cas system in biological research, and its influences and improvements remain a hot topic.

Mutations generated by CRISPR/Cas system can be accumulated over cell divisions, and in combination with the advantages of next generation sequencing (NGS) technology, lineage relations between ancestral progenitors and descendants can be traced based on their mutation patterns. This approach allows us to record lineage information through developmental processes, and drew a lineage tree based on the information, which provides a good intuition of where particular cells are originated from and where they go (McKenna et al., 2016): it offers a roadmap of cell development on a specific time scale.

Gene knock-down and activation systems employ the nuclease-dead Cas enzyme (dCas9), the endonuclease activity of which has been destroyed due to point mutations in the HNH and RuvC domains, to manipulate gene expression *in vivo* (Gilbert et al., 2013; Maeder et al., 2013). The dCas9/sgRNA functions as a sniper, with versatile bullets such as transcriptional repressors or activators, aimed at the target site. If dCas9 carries repressors (KRAB), it will decrease target gene transcription; and if dCas9 carries activators (VP64 or VP128) or modified sgRNA with MS2-binding site, it will increase expression of its target. Another way to achieve gene knock-down is by CRISPR/Cas13—an RNA-guided RNA-targeting system (Abudayyeh et al., 2017). CRISPR/Cas13 can cleave target transcripts *in vivo* with comparable efficiency and improved specificity using its own sgRNA for guidance. In addition, catalytically inactive Cas13a retains its RNA binding activity, and fused with florescent proteins, allows RNA movements in living cells to be tracked.

Fused with florescent proteins, dCas9 can be used to visualize the dynamics of coding and noncoding segments of DNA within the host genome (Chen et al., 2013). For telomeres (which contains large amount of self-repeated sequences), only one or a few sgRNAs are needed; for DNA segments without repeated sequences, more sgRNAs are required to visualize chromatin *in vivo*. Improved signal/noise ratio by splitting fluorescent protein or using the SunTag system, makes this imaging system more adaptive and practicable (Tanenbaum et al., 2014). Overall, the convenience and accuracy of this method eclipse that of traditional fluorescence -*in situ*-hybridization (FISH) method.

Laboratory evolution systems have great potential to generate enhanced genes, such as brighter florescent proteins and higher efficiency Cas enzymes. By coupling a nicking variant of Cas9 (nCas9) with an error-prone DNA polymerase, researchers have acquired EvolvR—an ideal targeted mutagenesis tool (Halperin et al., 2018), which allows to continuously mutate a target gene with a wide editing window. In addition, CRISPR-X and dCas9-AIDx systems have also been developed to perform gene evolution. Unlike EvolvR, these two utilize coupled cytidine deaminase to generate mutations within a limited editing window (Hess et al., 2016; Ma et al., 2016).

Epigenetic editing has been established as an essential tool for gene regulation. dCas9 fused with the core catalytic domain of the human acetyltransferase p300 or lysine demethylase 1 can increase or decrease histone H3 (H3K27) acetylation of the target site, resulting in expression alterations (Thakore et al., 2016). Also, Dnmt3a or Tet1 fused dCas9 can increase or decrease CpG methylation levels near the target genomic site, respectively (Liu et al., 2016). These epigenetic manipulations could be used to investigate relations between epigenetic marks and gene spatiotemporal expressions in a series of key development transitions.

Base editing (BE) systems have been developed to tackle the task of correcting single base mutations in a genome, due to avoiding the uncertainty of NHEJ and low efficiency of HDR. Thus, BE systems provide a directional and highly efficient approach to correct disease-associated single nucleotide polymorphisms (SNPs). Currently there are two kinds of base editing systems: ABE for A·T-to-G·C editing and BE for C·G-to-T·A editing (Gaudelli et al., 2017; Kim et al., 2017; Ren et al., 2017a; Xue et al., 2018). The ABE system consists of an RNA adenosine deaminase fused nCas9 and a targeting sgRNA, and can introduce base switching more efficiently with a low rate of indel occurrence. The BE system uses cytidine deaminase fused nCas9 to achieve C·G-to-T·A editing, with higher efficiency but at the possible cost of causing genomic-scale mutations. The power of such tools has been harnessed to study gene functional regulation (including gain-of-function, increased or decreased gene activity) by inducing point mutations on a whole gene level (Li et al., 2018). Future improvements to fine-tune the efficiency and off-target editing will be crucial for clinical applications of these tools (Koblan et al., 2018; Zuo et al., 2019).

Perhaps the most extraordinary aspect of the CRISPR/Cas system is how it can transform medical research (Men et al., 2017; Zhang et al., 2017). In addition to the previously described genomic manipulation aspects, a new approach using Cas12a has emerged to detect infectious diseases (Chen et al., 2018). After the Cas12a-crRNA complex binds and cleaves target dsDNA specifically, its ability to cleave ssDNA is activated. When a cleavable fluorescent reporter-coupled ssDNA is provided, this system can detect the existence of first dsDNA cleavage event, i.e. the existence of target disease-related dsDNA. Meanwhile, various methods based on Cas13 have been developed with much higher accuracy and simpler diagnostic procedures (SHERLOCK, SHERLOCKv2, and HUDSON combined SHERLOCK for example) (Gootenberg et al., 2018).

Indeed, a tool as powerful, versatile and convenient as the CRISPR/Cas system is a gift to biological research. CRISPR/Cas system and its derived systems give us new ways to study the complexity of life (Fig. 1). Future studies will focus on improving its specificity and efficiency, minimizing its off-target side effects, and also developing new derivations and applications.
